# Prospects and Applications of Natural Blood-Derived Products in Regenerative Medicine

**DOI:** 10.3390/ijms23010472

**Published:** 2021-12-31

**Authors:** Joanna Wessely-Szponder, Joanna Zdziennicka, Andrzej Junkuszew, Michał Latalski, Michał Świeca, Tomasz Szponder

**Affiliations:** 1 Sub-Department of Pathophysiology, Department of Preclinical Veterinary Sciences, Faculty of Veterinary Medicine, University of Life Sciences, 20-033 Lublin, Poland; joanna.wessely@up.lublin.pl (J.W.-S.); joanna.michalska15@gmail.com (J.Z.); 2 Faculty of Animal Sciences and Bioeconomy, Institute of Animal Breeding and Biodiversity Conservation, University of Life Sciences, 20-950 Lublin, Poland; andrzej.junkuszew@up.lublin.pl; 3 Children Orthopaedic Department, Medical University of Lublin, 20-093 Lublin, Poland; 4 Department of Biochemistry and Food Chemistry, University of Life Sciences, 20-704 Lublin, Poland; michal.swieca@up.lublin.pl; 5 Department and Clinic of Animal Surgery, Faculty of Veterinary Medicine, University of Life Sciences, Głęboka 30, 20-612 Lublin, Poland; tomszpon@op.pl

**Keywords:** neutrophils, antimicrobial peptides, neutrophil-derived antimicrobial extract, neutrophil degranulation product, platelet-rich plasma, microvesicles, tissue repair

## Abstract

Currently, there are a number of therapeutic schemes used for the treatment of various types of musculoskeletal disorders. However, despite the use of new treatment options, therapeutic failure remains common due to impaired and delayed healing, or implant rejection. Faced with this challenge, in recent years regenerative medicine started looking for alternative solutions that could additionally support tissue regeneration. This review aims to outline the functions and possible clinical applications of, and future hopes associated with, using autologous or heterologous products such as antimicrobial peptides (AMPs), microvesicles (MVs), and neutrophil degranulation products (DGP) obtained from circulating neutrophils. Moreover, different interactions between neutrophils and platelets are described. Certain products released from neutrophils are critical for interactions between different immune cells to ensure adequate tissue repair. By acting directly and indirectly on host cells, these neutrophil-derived products can modulate the body’s inflammatory responses in various ways. The development of new formulations based on these products and their clinically proven success would give hope for significant progress in regenerative therapy in human and veterinary medicine.

## 1. Introduction

Certain blood-derived products of autologous or heterologous origin are potentially useful for enhancing the healing process in veterinary and human medicine [1]. They were introduced for tissue repair/regeneration applications in the second half of the 20th century [2]. Therapies based on these factors mainly involve platelet-rich blood-derived concentrates or plasma concentrates rich in growth factors. These products are used in the treatment of osteoarticular disorders [3], osteoarthritis [4], corneal epithelial defects [5], tendinopathy [6], and other diseases [2,7]. Neutrophil-derived products have also been described as useful in a variety of medical applications, such as bone defect repair [8], treatment of periodontal tissues [9], enhancement of dental pulp stem cell differentiation [10], treatment of knee osteoarthritis [11,12], and some other musculoskeletal disorders [13]. For example, Liu et al. revealed that human antimicrobial peptide used in combination with bone morphogenic protein 2 promoted calvarial repair in a mice osteolytic bone defect model by acting on modified mesenchymal stem cells (MSCs) [14].

Neutrophils are highly reactive and short-lived cells because the inflammatory response must be prompt and robust. For this reason neutrophils are frequently involved in host tissue injury during excessive uncontrolled inflammation [15]. In such cases neutrophil-targeted therapeutic strategies could suppress unnecessary neutrophil activity either by reducing the number of neutrophils or by inhibiting their function [16]. Hence the medical interest in some autologous or heterologous compounds, including neutrophil-derived products consisting of bioactive peptides, and in their possible application as modifiers of neutrophil activity. They act directly on microorganisms by damaging or destabilizing bacterial, viral, or fungal membranes. These compounds are also involved in a plethora of innate immune and inflammatory responses, thus holding considerable promise for clinical use [17]. 

The evaluation of blood-derived products involves all the stages of translational research, including in vitro [9,14,18,19] and in vivo [5,20] studies, clinical veterinary [21,22] and clinical human studies [2,3,4,23,24]. Among the many different therapies based on blood components, special consideration should be given to the application of neutrophil-derived products. A number of them are being introduced for clinical use [17,25,26,27]. In this review, we discuss recent developments in the different autologous or heterologous products obtained from blood and their existing and potential biomedical applications.

## 2. Antimicrobial Peptides (AMPs)

### 2.1. The Origin, Classification, and Properties of AMPs

**Figure 1:**
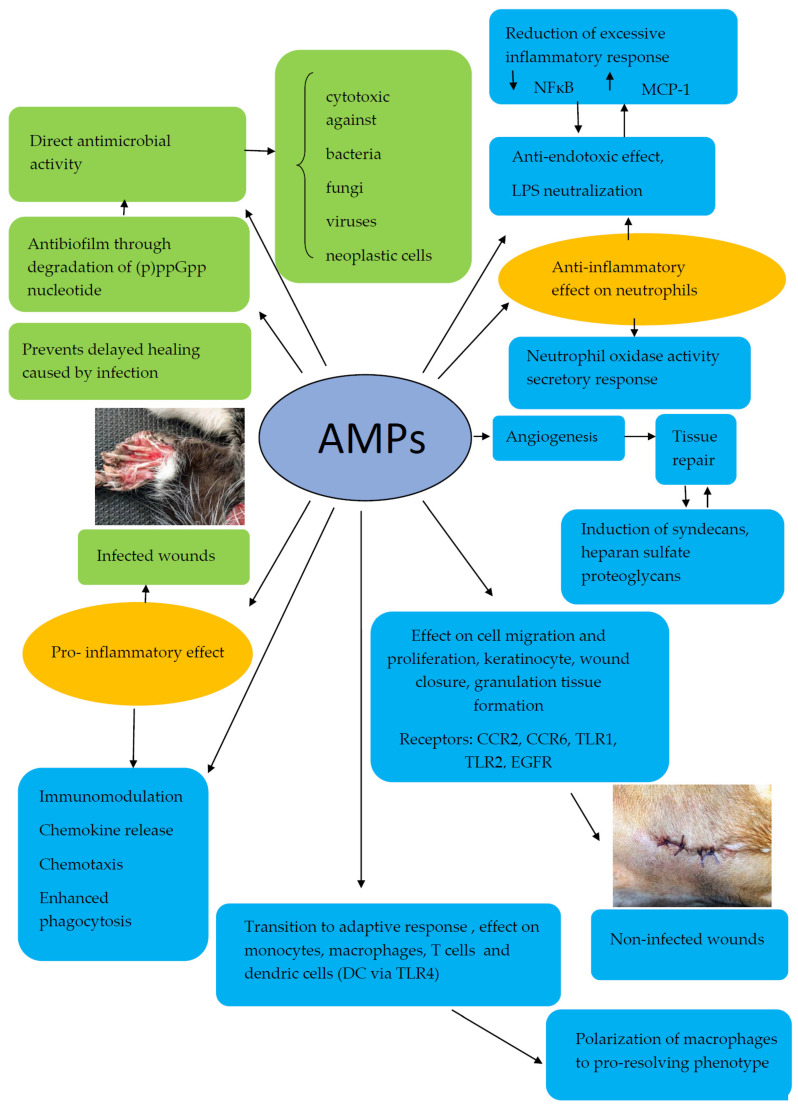
The pro- and anti-inflammatory effects of AMPs on different antimicrobial/cytotoxic activities (shown in green) and immunomodulatory/tissue repair functions (shown in blue).

Numerous antimicrobial peptides (AMPs) from humans, animals, plants, bacteria, and fungi have been isolated and evaluated over the past decades. Many different cationic antimicrobial peptides with varying compositions, structures, and distributions among species have been found in domesticated animals. Antimicrobial peptides generally are small polypeptides with short amino acid sequences, and less than 100 residues, that display antimicrobial activity [17,28]. AMPs appear to be important elements of the immune response against a broad spectrum of microorganisms [29]. These peptides, also known as host defense peptides, act as the first line of defense against microbes, thus playing a significant role in the innate immune system. AMPs display a broad spectrum of biological activities, including antibacterial, antifungal, antiviral, antibiofilm, and anticancer effects. They play major roles in chemokine induction, chemotaxis, inflammation, and wound healing. Peptides are also capable of enhancing phagocytosis, stimulating prostaglandin release, neutralizing the septic effects of lipopolysaccharide (LPS), promoting the recruitment and accumulation of various immune cells at inflammatory sites, stimulating angiogenesis, and inducing tissue repair [30,31,32,33]. Mammalian AMPs appear to play a key role in the transition to the adaptive immune response by acting on monocytes, macrophages, and T cells, and by exhibiting adjuvant and polarizing effects in relation to dendritic cell development [17,30] (Figure 1). 

There are two main groups of AMPs—cathelicidins and defensins. Defensins are classified as α-, β-, and θ-defensins. α-defensins 1–4 are present in the granules of neutrophils, and α-defensins 5 and 6 are predominantly found in Paneth cells. β defensins (hBD) have been detected in different tissues. The constitutive expression of hBD 1 to 4 has been identified in skin epithelium, and in the respiratory and urogenital tracts. hBD-5 and hBD-6 expression has been found in the epididymis. The expression of hBD-2, hBD-3, and hBD-4 increases after stimulation with microorganisms, inflammation, and during tissue injury and wound healing. In addition to their antimicrobial properties, hBDs serve a pro-inflammatory function by enhancing the production of cytokines/chemokines, which are involved in the pathogenesis of various inflammatory disorders. hBDs are chemoattractants for different cell populations, stimulating cell proliferation and angiogenesis during wound healing. Recent reports have demonstrated that hBD-2 and hBD-3 bind to and neutralize LPS, and hBD-3 also inhibits neutrophil apoptosis. These immunomodulatory properties of hBDs are associated with various receptors, such as chemokine receptors CCR2 and CCR6, toll-like receptors TLR1 and TLR2, and the epidermal growth factor receptor (EGFR)—which act via the signal transducer and activator of transcription (STAT) to improve wound healing, keratinocyte migration and proliferation—as well as G-protein-coupled receptors (GPCRs) [33]. Additionally, some defensins (human hBD-2) can suppress inflammation by influence on dendric cells via a TLR4-dependent mechanism together with activation of Th1 response [34].

Another group of AMPs are cathelicidins. As well as having direct antimicrobial properties, they serve multiple functions related to tissue repair and innate immunity. These peptides can be considered as potential replacement for antibiotics that have become ineffective due to microbial resistance, providing a much needed alternative. The immunomodulatory effects of these peptides—especially on immune cells, inflammation regulation, and repair processes in different tissues—are currently of considerable interest. Cathelicidins are effector molecules of innate immunity with diverse functions, including antimicrobial activity, modulation of wound repair, and inflammation by acting on inflammatory cells, among other macrophages. Macrophages, as active phagocytes involved in the elimination of pathogens, can exhibit pro-inflammatory properties. However, when the infection is resolved, they may change their function to resolve inflammation and promote tissue repair. Some reports have noted that AMPs are involved in regulating the polarization of these immune cells [35]. According to Pinheiro da Silva et al. [36], the effects of cathelicidins on macrophage response vary depending on the exogenous or endogenous origin of the peptide and prior cell activation. Hence cathelicidins can be used to modify the phenotype and function of tissue macrophages to promote the healing process.

### 2.2. Extensive Functions of AMPs in Wound Healing and Their Potential Applications

Wound repair is a dynamic multistep process that involves three stages: the first inflammatory stage; the second stage involving the formation of new granulation tissue, including connective tissue formation and angiogenesis; and the last stage—wound contraction and extracellular matrix (ECM) reorganization. Since wounds are an ideal breeding ground for microorganisms, proper wound repair requires maintaining a manageable microbial burden. Conversely, conditions that promote bacterial growth may result in chronic wounds that require long-term antimicrobial therapy [37]. 

The importance of natural AMPs in all stages of wound healing has been stressed by some authors [26,33]. The initial observation that LL-37 (a 37-amino acid peptide derived by proteolytic cleavage from the 18-kDa human cathelicidin antimicrobial protein, hCAP18) was abundantly present in acute wounds, but not in chronic wounds, provided the evidence that this peptide may be involved in natural wound healing. Further experiments on animal models and on an ex vivo model of human acute wounds confirmed the role of human cathelicidin as a potent stimulator of wound healing. 

In addition to natural AMPs, some synthetic analogues based on sequence motifs responsible for immunomodulation have been developed recently. Their mechanism of action is involved in modulation of specific intracellular signalling pathways, enhanced chemokine production and decreased release of pro-inflammatory cytokines, such as TNFa. Among these synthetic peptides, IDR-1018 (bovine cathelicidin bactenecin derivative) was selected due to its in vitro potential for wound closure and chemokine release. Additionally, this peptide showed lower cytotoxicity than LL-37 against skin cells in vitro. In a murine model, the effect of treatment with a 30 ng/dose of IDR-1018 was similar to this after application of 100-fold higher doses of LL-37. Higher concentrations of IDR-1018 (0.3 or 3 mg doses) significantly increased re-epithelialization in comparison with the same dose of LL-37. Histological analysis of wounds treated with IDR-1018 revealed that this peptide influences keratinocyte proliferation/migration [38].

AMPs can also support the formation of new blood vessels. LL-37 induces angiogenesis by increasing the vascular endothelial growth factor (VEGF) and by forming new capillaries [10]. Cathelicidin-related antimicrobial peptide (CRAMP), found in mice and similar to LL-37 in humans, is also involved in wound vascularization [39].The other animal-derived cathelicidin PR-39, a proline- and arginine-rich peptide isolated from porcine blood, is a potent angiogenic factor that inhibits ubiquitin proteasome-dependent degradation of hypoxia-inducible factor-1α protein, promoting vascularization, as revealed in a mice model [40]. Additionally, PR-39 can limit cell injury during inflammation by limiting NADPH oxidase-induced superoxide generation [41,42]. It also induced expression of syndecans, heparin sulfate proteoglycans with potential in wound repair [38].

In the course of re-epithelialization, cell migration and proliferation are important processes for adequate wound contraction, and some synthetic peptides increased the contraction capacity of human dermal fibroblast cells [43]. Other peptides can induce the synthesis of ECM components, especially collagen, by stimulating the differentiation of fibroblast to myofibroblast [26]. As estimated by Ramos et al., synthetic and recombinant LL-37 supported wound repair in a sterile wound model in dexamethasone-treated mice by increasing vascularization and re-epithelialization [44]. Some peptides enhance re-epithelialization by activating receptor-signaling pathways for cell migration/proliferation (phospholipase *C*-beta (PLC-*β*), EGFR, and phosphoinositide-3 kinase (PI3K)/Akt/mechanistic target of rapamycin (mTOR) pathway). The mechanism of action through EGFR transactivation to promote cell migration has been described both in natural AMPs, such as LL-37 and melittin, and in synthetic peptides [26,43,45]. EGFR transactivation is mediated by the cleavage of membrane-bound EGFR ligands by metalloprotease, leading to ERK1/2 phosphorylation or STAT3 phosphorylation and keratinocyte migration and/or proliferation, resulting in the activation of the ERK1/2 pathway, which promotes keratinocyte migration and/or proliferation [26,43,45]. The key role of purinergic receptor activation (P2X7 receptor) and mitogen-activated protein kinase (MAPK) in EGFR transactivation has been underlined. In an earlier study, human catestatin activated the EGFR-coupled PLC-*β* pathway and stimulated the phosphorylation of the MAPK family, including ERK and p38, and thus promoted both cell proliferation and migration. This mechanism suggested a crucial role of the P2X7 receptor, intracellular calcium, and reactive oxygen species (ROS), although whether the peptide induces the activation of the P2X7 receptor indirectly or by acting as an allosteric modulator remains unclear. It is noteworthy that some peptides such as LL-37 promote only cell migration, while others such as melittin stimulate both proliferation and migration. This phenomenon indicated that cell proliferation and migration may be mediated by different mechanisms after EGFR transactivation [43].

All these findings clearly indicate that peptides from diverse sources are upregulated at all stages of wound healing, promoting cell migration, proliferation, and angiogenesis, hence the assumption that these peptides markedly contribute to the healing process and thus, show promising application prospects in the field of regenerative medicine [43].

### 2.3. Applications in Treatment of Chronic, Infected, Hard-to-Heal Wounds

Delayed or impaired wound healing can be caused by bacterial contamination and colonization, and most wounds are contaminated by bacteria. AMPs designed for the treatment of infected wounds should display low cytotoxicity and a broad spectrum of antimicrobial activity. Additionally, therapeutic AMPs should stimulate wound closure and remain stable under different conditions in the host environment, especially at high salt concentrations and in the presence of proteases released in the wound site from the host cells or by invading bacteria [46]. It should be mentioned that more than 100 endogenous proteases have been found in wound fluid—in particular metalloproteases and neutrophil elastase, but also proteases from bacteria. The synthetic peptide SHAP1 seems to be a promising therapeutic option because of its high stability in the presence of proteases and high salt concentrations. This promotes wound healing in vivo at the same low concentrations used in vitro [46]. To decrease proteolytic degradation, some synthetic peptides, such as Novispirin G10, have been developed for rapid bacterial killing. This peptide has been found to kill bacteria within four hours after intradermal injection into a burn wound infected with *P. aeruginosa* [45]. Furthermore, the rapid bactericidal activity of AMPs reduces treatment duration compared to conventional antibiotics, and lowers the potential for developing resistance [47]. 

Different mechanisms are involved in antimicrobial properties of AMPs, as in the case of the lactoferrin-derived peptide HLR1, which acts against *S. aureus* in an ex vivo skin model. However, some peptides protect against infection without direct antimicrobial properties, based only on their immunomodulatory properties. Additionally, infections with *S. aureus* are often associated with an excessive inflammatory process, resulting in higher severity of infection. Thus, anti-inflammatory properties of AMPs might be beneficial, especially in the healing of chronic and hard-to-heal wounds such as diabetic leg ulcers. The peptide FI-PRPRPL-5 enhanced wound closure in a mouse model of skin infection. It is also capable of reducing the expression of pro-inflammatory cytokines, such as TNF, IL-1ß, and IL-6. The peptide ca-Tx-II also acts as an anti-inflammatory by inhibition of NF-κB activation and thus release of pro-inflammatory cytokines together with increase of mediators involved in wound healing such as MCP-1. The peptide DIK8 via topical application significantly reduced bacterial growth in a mouse model, and decreased release of pro-inflammatory cytokines. The innate defense-regulator peptide (IDR-1) modified signaling pathways involved in TLRs, therefore upregulating expression of chemotactic mediators for monocytes/macrophages and downregulating pro-inflammatory cytokines. The cathelicidin HDP fowl-1 protected mice from MRSA infection by enhancing the host response by activation of inflammatory cells, such as macrophages and neutrophils [45]. The peptide IDR1018 enhanced wound healing in a porcine model infected with *S. aureus* as well as in non-diabetic mice. Conversely, the antimicrobial effect of IDR-1018 was not observed in diabetic mice. The possible explanation is assumed to be that the compromised immune system in diabetic animals blocks one or more signaling pathways by which IDR peptides exert their effects [26].

In patients suffering from chronic leg ulcers, clinical trials revealed that supplementation of LL-37 to non-healing wounds significantly increased the healing rate. Accordingly, a phase IIb clinical trial has been commenced to determine the dose-response efficacy of LL-37 in patients with nonhealing venous leg ulcers [25]. Apart from human cathelicidin, there are some mammalian AMPs proposed for clinical applications. The acid-pepsin digestion of bovine lactoferrin results in the release of the peptide lactoferricin, which shows the strongest antimicrobial activity among mammalian lactoferricins and has potent immunological properties [48]. The bovine lactoferrin derivative, a small peptide of 25 amino acids (LFcinB), has antimicrobial effects against Gram positive and Gram negative bacteria, as well as immunomodulatory properties. It improves diabetic wound healing by causing angiogenesis and collagen deposition, and by reducing inflammation [49]. 

Additionally, the anionic peptide dermcidin from human neutrophil granules of humans displays a good spectrum of antimicrobial activity for possible treatment of infected skin wounds [48].

Like cathelicidins, defensins also induce cell migration and proliferation, as well as promote angiogenesis and vascularization. An increased concentration of these peptides at the wound site is mediated in part by cytokines and growth factors. In animal models, the topical application of hBD-3 stimulated wound closure in infected diabetic wounds. Increased production of defensins, including hBD-2 and hBD-3, was observed during the proliferative stage of wound healing. Moreover, hBD-2 expression was induced in chronic venous ulcers, whereas hBD-2 and hBD-3 were downregulated in diabetic wounds. Similarly, significant decreases in hBD-2 expression were observed in burn wounds, which could explain the increased susceptibility to infection and sepsis in burn patients [33]. These peptides also possess antimicrobial activity as an integral part of their positive influence on wound repair. However, especially in case of hBD-3, therapeutic applications of these peptides is limited due to their large size, and thus high associated costs of production, as well as their sequence complexity (disulfide bridges) and high cytotoxicity [45].

These observations suggest that different AMPs show various effects under different clinical conditions, which makes them promising therapeutic agents to improve the healing of miscellaneous wounds [33]. This multifactorial mechanism of action is very powerful, and involves the minor tendency of AMPs to evoke microbial resistance. This makes AMPs especially attractive candidates, possibly better than conventional antibiotics, for the local treatment of a disturbed healing process. Importantly, although resistance or cross-resistance to AMPs has been described to develop in vitro, no comparable results have been found in vivo. The likely reason is that the development of AMP-resistant strains is much slower than under in vitro conditions and the host/endogenous AMP interactions are essential for this mechanism [50]. 

Different mechanisms of action by AMPs could be used in the treatment of different kinds of wounds. In the healing of non-infected wounds, the effects on skin cell migration and proliferation appear essential, whereas antimicrobial and immunomodulatory properties might be used to treat infected wounds [26]. However, the long-term therapeutic use of exogenous AMPs should be carefully considered before introduction into broad clinical practice because of the risk of side effects and possible compromising of the innate immune defense [50].

### 2.4. Healing of Burn Wounds

Some AMPs appeared to be good candidates to treat burn wounds. One LL-37 analog, the synthetic LLKKK18 peptide, displays higher hydrophobicity and cationicity than LL-37. This results in higher antimicrobial and chemoattractant activity. Moreover, conjugation with dextrin stabilizes the peptide and protects it from proteases, especially in a burn tissue environment. The dextrin-LLKKK18 conjugates prepared as a Carbopol hydrogel for topical administration released LLKKK18 from dextrin conjugates and improved healing of burn wounds. This was achieved through modulation of the oxidative stress and a faster resolution of the inflammatory phase by recruiting M2 macrophages early post-injury. Moreover, it stimulated angiogenesis in the granulation tissue, which provides the rapid transport of growth factors, cytokines, and chemokines to the site of injury, thus contributing to a faster healing with proper tissue organization. Carbopol is made of carbomers that are cross-linked to form a structure with good buffering capacity, high viscosity, bioadhesive properties, and thermal stability. This conjugate has been described as a safe, biocompatible, inexpensive, and effective formulation for the treatment of burn wounds, which is easy to apply and remove, maintains a moist environment, and possesses antimicrobial properties. Therefore, this delivery system has great potential as a therapeutic approach for treatment of burn wounds [51].

### 2.5. Other Functions of AMPs in the Repair Process

Some findings demonstrate that AMPs have the potential to promote osteogenesis and osteoclastogenesis, especially in inflammation-induced bone loss caused by such disorders as periodontitis and rheumatoid arthritis. The complex of LL-37 with MSCs stimulates the formation of new bone through the stimulation of angiogenesis and seems to be a potential new therapeutic option for the treatment of bone loss involved in inflammatory conditions [8,14]. Neutrophil-derived AMPs have also been applied in the treatment of bone infections [52]. The synthetic variant of murine cathelicidin CRAMP-derived peptide, with a broad spectrum activity against various Gram positive and Gram negative bacteria, is a promising compound in the development of biofilm-preventive coating of bone implants [32]. Some authors underlined the role of LL-37 in the regeneration of the dentin-pulp complex by promoting the migration of MSCs of the apical papilla in immature permanent teeth. This mechanism provides a suitable environment for angiogenesis and inhibits bacterial proliferation. A study by Milhan et al. suggests that LL-37 induces cell proliferation and may contribute to the differentiation of dental pulp stem cells into odontoblast-like cells. This makes it a possible adjunct for the regeneration of immature permanent teeth after pulpal necrosis [10]. Furthermore, LL-37 has induced the migration, proliferation, and wound closure of airway epithelial cells, whereas both hBD-3 and LL-37 stimulate corneal epithelial cells to enhance ocular surface repair [53].

Another AMP, synthetic peptide PXL01, derived from the iron-binding glycoprotein lactoferrin, has shown phase II clinical trial efficacy for the prevention of post-operative adhesions in patients after flexor tendon repair surgery. Under in vitro conditions, PXL01 suppressed the most important adhesion markers by reducing pro-inflammatory cytokine secretion while promoting fibrinolysis. Additionally, PXL01 efficiently reduced post-surgical adhesion formation in experimental models of abdominal surgery in rats and during flexor tendon repair surgery in rabbits. Notably, no toxicity to host cells was confirmed in vitro and no negative effects of PXL01 treatment were observed after experiments in animal models, specifically on the healing of bowel anastomosis of rats or the repaired tendons in rabbits. In view of these findings, a phase III clinical trial could be conducted to address the dose-response efficacy of PXL01 in reducing adhesion formation and improving post-surgical recovery [25].

Articular cartilage has been noted for its ability to topically generate some antimicrobial peptides when challenged with microorganisms. The expression of hBD-2 in microbicidal doses suggests that antimicrobial peptides may contribute to host defense mechanisms in joints. Human and murine articular cartilage holds potential for reducing infection through the endogenous production of antimicrobial peptides. Moreover, it proves the existence of innate immune defense mechanisms with the potential to support the repair process [11]. Additionally, the lactoferrin-derived LFcinB peptide exhibits potent anti-catabolic and anti-inflammatory activity in human articular tissues, and is applied in the treatment of osteoarticular disorders [54].

Neutrophil-derived extracts containing a mixture of different AMPs were initially used as antimicrobial natural products obtained from the blood of farm animals [28,41,55,56,57]. However, in addition to their well-known antimicrobial activity, these blood-derived products have also been proven to exhibit immunomodulatory and tissue repair properties, enabling their wider application in regenerative medicine. A crude neutrophil extract, obtained during isolation from neutrophil granules and lyophilized, is resistant to long-term storage and could be used as an autologous or heterologous product to promote the healing process in different tissues [58,59].

Neutrophil extracts differ in composition depending on animal species. When obtained from isolated porcine neutrophils, they contain the largest variety of cathelicidins, including PR-39, prophenins, and protegrins. However, they lack defensins [57,60]. Ovine neutrophils contain cathelicidins such as SMAP-29, SMAP34, OaBac5, OaBac6, Oabac7.5, and OaBac11 [41]. In a rabbit neutrophil extract, mass spectral analysis confirmed the presence of the rabbit 15-kDa antibacterial protein, as well as defensins NP-1, -2, -3a, -3b, -4, and -5 [61]. Some in vitro studies indicated that the addition of an AMP extract into neutrophil and macrophage cultures changed the activity of inflammatory cells obtained during biomaterial implantation in an animal model [30,58,59]. After implant insertion, monocyte-derived macrophages (MDMs) are among the first cells at the implant site. They are considered key regulators of both the initiation and the resolution of inflammation [62]. Therefore macrophage stimulation with an AMP extract could be used for the enhancement of tissue repair and remodeling, especially for patients with impaired healing and regenerative capacity. Macrophage phenotype modification provides a new strategy to obtain a safe and effective method to improve the healing of different tissues with a reduced risk of complications [30,58]. According to Mansour et al., macrophages stimulated with the synthetic peptide IDR-1018 developed a unique phenotype with the ability to maintain critical pro-inflammatory activities, simultaneously releasing anti-inflammatory and regulatory mediators [63]. Pena et al. found that this peptide promotes wound healing by stimulating M2-differentiated macrophages to increase the expression of different healing genes that are critical for tissue repair [64].

In addition to macrophages, the repair process also importantly involves neutrophils. These cells are involved in removing cellular debris at the injury site and release compounds—including growth factors or proangiogenic factors stored in their segmented nuclei and granules—that attract monocytes and differentiate them to macrophages [65]. The role of neutrophils in bone fracture healing is associated with the inflammatory, repair, and remodeling stages. At the initial inflammatory stage, neutrophils are found in abundance in the fracture hematoma [66]. Within the first 48 h after injury, they contribute to fracture healing by synthesizing ECM with fibronectin [67]. Because of the multifunctional role of neutrophils, their stimulation or suppression seems to be critical for tissue repair [66]. Accordingly, efforts have been made to regulate their secretory response using AMP extracts. When stimulated with an autologous AMP extract, rabbit neutrophils exhibited reduced activity in terms of their enzymatic response, as well as decreased free-radical generation before and after biomaterial implantation [59]. These findings corroborated previous studies on the anti-inflammatory effects of PR-39 [68], and also indicated that AMPs have the potential to regulate neutrophil inflammatory response.

### 2.6. Antibiofilm Activity of AMPs in Prevention and Treatment of Biomaterial-Associated Infections

Biofilm formation by bacteria such as *S. aureus*, *P. aeruginosa*, and *Streptococcus*, is a critical issue in treatment of infectious diseases. The biofilms can induce and maintain chronic inflammation in the wound site and disturb infected wound healing, causing inhibition of the migration and proliferation of epithelial cells. The treatment needs high doses of antibacterial agents for topical application because of hindered penetration through the biofilm. If the treatment is incomplete, the residual biofilm can gradually recover within 24 h after debridement. Since AMPs appear to be molecules with high activity against a broad spectrum of bacteria, they could be applied against biofilm. Their good destructive and inhibitory effect against biofilms is based on the ability to degrade nucleotide, guanosine penta- and tetraphosphate (p)ppGpp, which play a vital role in biofilm development. Additionally, some AMPs (IDR-1018) can be utilized to prevent and kill bacterial biofilms through synergistic interactions with antibiotics [34,53].

Severe infections with biofilm formation are associated with implant insertions and use of biomedical devices, due to their high susceptibility to bacterial colonization. Frequently, implant surfaces become a reservoir of bacteria that can spread into the whole body, which leads to persistent chronic infections [53]. For these reasons, biomaterial-associated infections remain a serious global problem, especially in orthopedic applications of titanium (Ti) implants. Such infections may occur after surgery, particularly at the initial acute infection stage. At this stage, bacteria aggregate on the implant surface and form bacterial biofilms to protect themselves against host immune defenses and environmental factors. Biomaterial-associated infections have serious consequences, including implant failure, disability, sepsis, and even death [69]. These infections are difficult to treat and in most cases require implant removal and long-term antimicrobial therapy [52].

In view of the growing resistance to conventional antibiotics, and with implant-associated infections being among the most feared complications in orthopedic surgery, there is an emerging need for the development of effective approaches to prevent the bacterial colonization of orthopedic implants. AMPs may be promising candidates here, due to their potent and broad-spectrum antimicrobial activity, their low potential to induce resistance, and their ability to remain effective in the immobilized state [70]. Therefore, some synthetic AMPs with potential against biofilm formation, such as AG-30, AG-30/5C, WRL3, melimine and Mel4, 73c, and D-73, seem to be a promising therapeutic option [53].

In one study, a titanium disc was combined with BMAP27(1–18), an α-helical cathelicidin antimicrobial peptide, to prevent bacterial infection of orthopedic implants caused by *Staphylococcus* species. This combination displayed antimicrobial properties against the reference strain of *Staphylococcus epidermidis* and biofilm-forming capability. A reduced bacterial count and morphological alterations of adhering cultures confirmed the rapid antimicrobial effect of this compound. What is more, the immobilized AMP was not cytotoxic to osteoblasts, which adhered and spread better on functionalized Ti. These results suggest that Ti functionalization with BMAP27(1–18) could be promising for the prevention of bacterial colonization in some bone graft applications [71].

Another treatment option involves the localized release of AMPs from implants. It has recently been proposed as an efficient antimicrobial approach. This mode of application ensures an adequate therapeutic concentration of antimicrobial agents at the target site, and can also display a minor systemic impact to reduce cytotoxicity to patients. Conversely, covalently immobilized AMPs show lower stability after contact with bacterial proteolytic enzymes and low activity against unattached bacteria. Thus the localized delivery of AMPs appears to be a better therapeutic option with better protection of the AMPs from proteolytic degradation, higher ability to kill the bacteria around the implant, and lower cytotoxicity than after systemic administration. Currently, the localized delivery of AMPs is usually achieved by loading AMPs on a substrate, such as calcium phosphate films, or through interaction between TiO_2_ and an anchor sequence. Another approach that has attracted considerable attention involves titania nanotubes (TiO_2_ nanotubes, Ti-NTs), which display a unique topography, high surface-to-volume ratio, and superior loading capability [69].

## 3. Neutrophil Degranulation Product (DGP)

Some authors have noted that during migration from the circulatory system to tissues, neutrophils sequentially release their different granule types to communicate with other immune cells, especially monocytes and macrophages. These granules are stored in four different compartments, and are sequentially released [72]. They are formed during myelopoiesis—primary granules at the stage of early promyelocyte, secondary granules in myelocytes and metamyelocytes, and tertiary granules in band cells. As well as having a direct antimicrobial effect, granule proteins are involved in ROS formation, macrophage activation, bacterial opsonization, and the recruitment of monocytes and macrophages [73,74]. In vitro studies conducted by Hussen et al. revealed that a product prepared from granule contents after the activation of bovine neutrophils could be used for the differentiation of MDM towards a mixed macrophage phenotype with enhanced antimicrobial functions [72]. More recent research [58] has shown that after the stimulation of ovine MDM with autologous DGP, macrophages show a partially pro-inflammatory signature characterized by intact arginase activity. This activity is especially useful for enhancing the repair process in bone and other tissues. These findings corroborate those of Hussen et al. regarding DGP influence on macrophage phenotype [72]. According to Soethlein et al., various stimulators caused the release of different products, having different effects on the phagocytic activity of monocytes and macrophages [73]. However, the products released from neutrophils are critical for neutrophil/macrophage interactions in tissue repair. Given the primary role of pro-resolving macrophages in tissue repair, macrophage modulation seems to be a useful therapeutic option [72].

## 4. Neutrophil-Derived Extracellular Vesicles in Regenerative Medicine

Many eukaryotic cells can release small vesicles, such as exosomes, ectosomes, microvesicles, shedding microvesicles, and microparticles. The release occurs either spontaneously or in response to various stimuli, as a mechanism for cell-to-cell communication [75]. Extracellular vesicles (EVs) consist of small exosomes (50–100 nm). These exosomes are released from a multivesicular body and larger vesicles called microvesicles (MVs), originating directly in the cell membrane [75,76]. Depending on their cellular origin, EVs are involved in a broad spectrum of biological activities. These molecules can mediate cell-to-cell communication in many physiological and pathological processes, including antigen presentation, immune signaling, angiogenesis, aging, and cell proliferation and differentiation [76]. MVs are involved in different diseases such as atherosclerosis, thrombosis, rheumatoid arthritis, cancer, lung injury, and sepsis. This makes them a promising solution for both therapeutic and diagnostic applications [77].

Following their successful applications in oncology and immunology, some authors have noted the potential of EVs in regenerative medicine [76,78,79]. EVs are involved in restoring tissue and organ damage, and their paracrine effects have been observed in stem cell-based therapeutic approaches.

**Figure 2:**
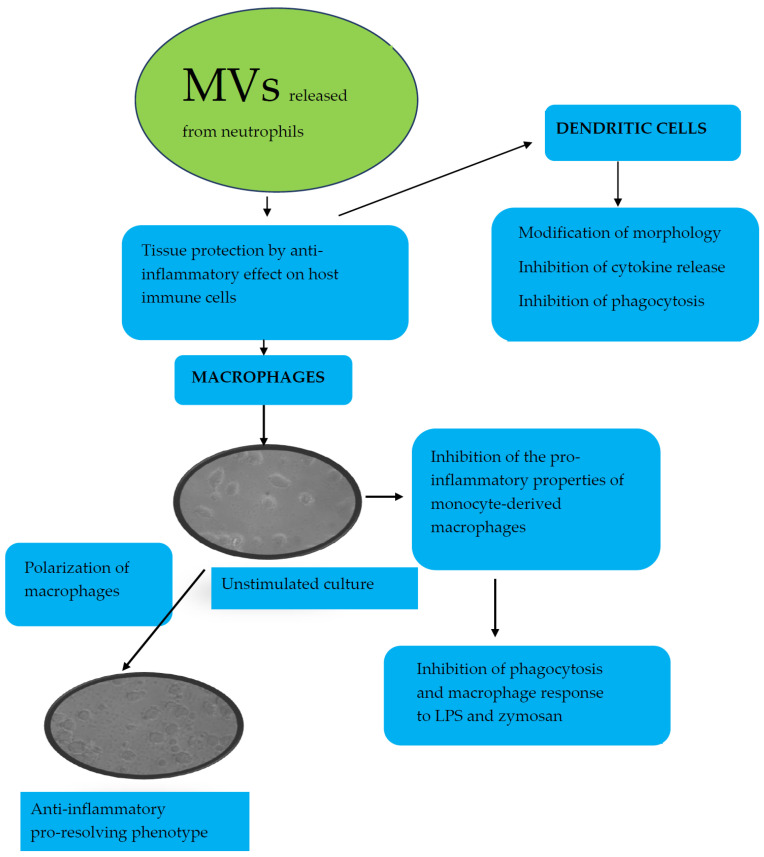
Different effects of neutrophil-derived MVs on host cells, especially on monocyte-derived macrophages and dendric cells.

In this review, we have focused on neutrophil-derived products. It should be emphasized, therefore, that EVs are also released in the form of MVs from activated neutrophils during degranulation. Neutrophil-derived MVs are right-side-out structures with cytosolic content and a diameter of 50–200 nm. The composition of MVs, which contain lipids, proteins, and nucleic acids, depends on their origin and way of activation [78,80]. MVs are known to promote tissue protection and repair by acting on some inflammatory cells. Unlike other MVs, neutrophil-derived MVs have recently been shown to inhibit the pro-inflammatory properties of monocyte-derived macrophages under in vitro conditions [59,78,80]. Overall, neutrophil-derived MVs have anti-inflammatory or inflammation-resolving effects on cells of a myeloid lineage, inhibiting phagocytosis and macrophage response to LPS and zymosan [77]. A recent study has confirmed that the stimulation of monocytes with neutrophil-derived MVs causes the polarization of MDM towards an anti-inflammatory pro-resolving phenotype [58]. This effect could be explained by both the induction of TGF-1 secretion from MDM and the presence of phosphatidylserine on the outer leaflet of the MV membrane. Moreover, neutrophil-derived MVs influenced dendric cells, causing modification of their morphology, inhibition of release of pro-inflammatory cytokines and phagocitic activity, and these effects were also ensured by stimulation with TGF1ß [78] (Figure 2).

## 5. PRP and Platelet/Neutrophil Co-Culture Interactions

Some naturally occurring products, referred to as “autologous biomaterials”, are used for stimulating bone and soft tissue healing and resolving infection. Platelet-rich plasma (PRP), a preparation of concentrated autologous platelets containing many bioactive factors, is one such biomaterial that has been widely used in clinical practice. Platelet concentrates and other platelet-related products are among the most studied blood-derived products applied in regenerative medicine. However, there are many protocols to obtain PRP with different contents, with or without leukocytes, and one standardized method is still unavailable [81].

PRP has been used in animals and humans to treat many disorders in dentistry, neurosurgery, cardiothoracic and maxillofacial surgery, orthopedics, and traumatology [82]. Platelets are important modulators of inflammation; they express some toll-like receptors, and can enhance leukocyte effector functions, including pro-inflammatory activity [83]. This pro-inflammatory effect has been particularly noted in PRP rich in leukocytes, since this combination enhances some functions of neutrophils and activated platelets [7,84]. Some studies described reciprocal in vitro interactions between platelets in PRP and neutrophils in co-cultures of these cell populations. Gros et al. noted that activated platelets have the potential to regulate neutrophil functions such as phagocytosis, ROS formation, neutrophil degranulation, or the formation of neutrophil extracellular traps [84]. The anti-inflammatory potential of the interactions between neutrophils and platelets was described by Lana et al. [12]. These authors noted that the tissue-repair effect of a combination of neutrophils and platelets obtained from pure PRP could be more beneficial than detrimental. There are two different mechanisms to explain these interactions. Once activated, platelets can release arachidonic acid, which is captured by neutrophils and used in the production of leukotriene and prostaglandins. Alternatively, platelets, together with neutrophils, can pick up and convert leukotrienes into lipoxin—a potent anti-inflammatory factor that reduces neutrophil activation and migration, initiating the resolution phase of the inflammatory process.

The different types of cellular response result from the different compositions of PRP. Some in vitro studies found that platelets can inhibit enzyme release from neutrophil granules [85]. Conversely, according to Gros et al., platelets increased lysozyme secretion by neutrophils stimulated with opsonized zymosan [84]. Likewise, ROS might play a key role in the complex interactions of neutrophils with platelets, as well as participate in communication between these cells. However, the detailed mechanism of these interactions remains unclear. The main lipid mediators involved in these processes are PAF, leukotriene 4, and tromboxane (TXA), released by platelets. By extension, it is possible that TXA2 affects the oxygen metabolism of neutrophils, which lack this enzyme. This suggests a significant role of platelets in the activation of neutrophils and could explain the molecular mechanisms of an excessive inflammatory process [86]. The anti-inflammatory effect of PRP seems to be based on the different mechanisms involved in reducing elastase secretion to prevent inflammatory host damage [15]. However, it should be noted that the pro- or anti-ROS effect of a platelet concentrate depends on the cause of inflammation, the number of activated leukocytes, platelets, and endothelial cells, as well as on such experimental conditions as the presence of platelet and neutrophil stimulators, and other environmental factors. Overall, the effects of platelets on the inflammatory process can either be beneficial or deleterious, depending on the wider pathophysiological context [84]. A number of studies have found that during the in vitro treatment of tendon cells, the primary role of PRP at the inflammatory stage of tissue repair is to release major growth factors, which have anti-inflammatory effects, and to reduce the release of pro-inflammatory cytokines. The presence of leukocytes in PRP could intensify inflammation because they increase the expression of IL-1*β*, IL-6, and TNF-*α* in tendon cells. A study on this model demonstrated that leukocyte-rich PRP could exacerbate inflammation in tendon cells, whereas pure PRP without leukocytes could have anti-inflammatory effects by decreasing IL-6 gene expression [6].

These different modes of action by activated platelets in concentrates of different contents have been used in regenerative medicine. Increased inflammation can be necessary to treat a chronic wound with impaired and delayed healing. The potential for angiogenesis and tissue restoration, in turn, is useful for bone healing. However, because of the destructive effects of overactivated neutrophils on different tissues, their addition should be avoided in some clinical applications. The presence of neutrophils in PRP can lead to pro-inflammatory stimulation [87]. In platelet/neutrophil co-cultures, previous neutrophil activation can cause an increase in their pro-inflammatory properties. Zhou and Wang [6] demonstrated that the use of PRP with neutrophils could lead to a higher collagen type III-to-collagen type I ratio, causing fibrosis and decreased tendon strength. Other neutrophil-mediated deleterious properties include the release of inflammatory cytokines and matrix metalloproteinases that promote pro-inflammatory and catabolic effects when applied to tissues [7]. Moreover, activated platelets have been found to play a key role in the development of biomaterial-associated inflammatory disorders by promoting the accumulation of neutrophils and enhancing their antimicrobial functions [88].

Overall, the immune functions of platelets vary depending on the combination of different stimuli received by the neutrophil/platelet population. These responses can cause either the augmentation or attenuation of neutrophil functions in the absence as well as in the presence of TLR stimulation with LPS [15].

## 6. Prospects and Limitations in Clinical Applications

It is worth stressing that there have been some advances in the therapeutic application of AMPs. These include a good safety and tolerability profile, a broad range of antimicrobial and immunomodulatory action, and a low potential for resistance development. However, there are certain issues and concerns associated with AMPs in clinical applications, such as their cytotoxicity against eukaryotic cells, immunogenicity, drug resistance, hemolytic activity, and other side effects. These peptides can have highly toxic side effects on mammalian cells in long-term use, and hence research is under way to increase the effectiveness and safety of AMP-based drugs [17]. Other problems involved in the therapeutical use of AMPs include their limited stability during storage at ambient temperatures, a short half-life due to high sensitivity to proteolytic degradation, and fast elimination involved in low oral availability. Physiological conditions, such as low pH, high ionic strength, and contact with blood components, may significantly suppress their activity. Other factors that reduce their availability include high manufacturing costs, especially for long peptides in combination with high doses and long-term administration, and the limited number of manufacturers that can produce peptides on a commercial scale [25,34] (Table 1).

Despite a large number of AMP clinical trials, only a few have achieved satisfactory results, especially in the field of regenerative medicine [43]. As examples, pexiganan (Locilex^®^), which was used for treatment of infections in diabetic foot ulcers, failed during clinical phase III. Iseganan (IB-367, protegrin-I analogue), used for preventing polymicrobial oral infections such as stomatitis and ventilator-associated pneumonia, failed in phase III, because it did not show significant efficacy. Recently, phase III trials of Murepavadin (POL7080), a protegrin analogue for intravenous treatment of bacterial ventilator-associated pneumonia, were terminated due to increased serum creatinine levels in patients, indicative of acute kidney injury. These failures were attributed to the lack of significant efficacy compared to other antibacterial drugs or to multiple adverse effects. However, phase III clinical trials using intravenously administered Brilacidin, a synthetic defensin analogue for treatment of skin infections, will start soon after successfully finishing phase II [27,48,53] (Table 2).

Legal provisions have also greatly delayed the clinical development of AMPs. The regulatory agencies require that the antibacterial effects of new antimicrobials should not to be worse than existing ones. Previously, some AMPs with good antibacterial properties (e.g., omiganan and pexiganan) encountered regulatory obstacles in clinical trials. However, due to reduced approvals of new anti-infectives and the increase in bacterial resistance to antibiotics, the regulatory agencies have taken new measures to promote the development of new anti-infectives, which give the clinical trial design market more flexibility [43].

Several natural and synthetic AMPs have shown high cytotoxicity to mammalian cells. Currently, there is no uniform or standardized method to evaluate the toxic side effect of AMPs, and the hemolytic activities reported as “safe” vary greatly between studies. Besides, due to the limited AMP clinical trials, the knowledge of adverse effects in humans is incomplete. To date, only a few publications have reported the standardized toxicology datasets for AMPs [43].

Some limitations in the therapeutic use of AMPs are associated with their properties as effective disruptors of biological membranes, resulting in cytotoxicity against host cells. To address these concerns, nanotechnology (encapsulation with suitable nanocarriers) has been used for the delivery of AMPs. AMP encapsulation techniques primarily involve the application of inorganic materials, polymeric carriers, and lipid-based approaches [89].

Gold nanoparticles (AuNPs) conjugated with LL-37 promoted wound healing by supporting collagen synthesis and degradation and having anti-inflammatory and angiogenic properties to reduce pro-inflammatory cytokines and increase VEGF and bFGF generation. Mice model studies corroborated enhanced wound healing with LL-37 AuNPs compared to AuNPs alone. This conjugate exhibited higher collagen and IL-6 expression and lower myeloperoxidase activity than LL-37 alone, indicating stronger anti-inflammatory and antioxidant properties compared to immobilized peptides [89].

AMP microencapsulation with Poly (lactic-co-glycolic acid) nanoparticles increased their immunomodulatory and pro-angiogenetic effects by protecting AMPs from degrading enzymes and facilitating their controlled release. Moreover, this encapsulation enhanced wound healing through the release of LL-37 and lactate [89].

Such formulations as cream and ointment possess some disadvantages: they can be easily removed from the wound site, which can limit the amount of AMP reaching the target. Furthermore, there is minimal or no control in AMP release and penetration, which strongly limits the treatment efficacy [90].

On the other hand, wafers provide an interesting approach using AMPs for topical application. However, the peptides need to be flexible to exhibit their antimicrobial activity and it might be challenging to achieve this property with the wafers. Suitable modifications, including the covalent conjugation of AMPs with the surface of the wafers using a flexible linker, can solve this problem. Such modified wafers covered with a secondary wound dressing such as OpSite can provide prolonged antimicrobial and tissue repair effects [90].

Lyophilized wafers—a novel delivery technique for infection control and wound treatment—are manufactured with mixed solutions, suspensions, or gels of natural polymers with AMPs. The most common polymers for wafer preparation are gums, including guar, xanthan, karaya, and polymers such as sodium alginate and carboxymethylcellulose. These wafers are directly applied onto the wound and their swelling and flow properties can be modulated by viscosity modifiers to provide sustained release of the antimicrobial factor. The wafers can provide controlled delivery of AMPs and absorb excessive fluid at the injury site, especially in suppurating wounds [89].

Hydrogels are drug delivery carriers of natural or synthetic polymers that can maintain a humid environment due to a high water content, which is essential for proper wound healing. These hydrogels can be functionalized with AMPs to provide antimicrobial activity, holding considerable potential as dressing materials for the treatment of infected wounds [89]. However, further optimization studies to maintain optimal therapeutic concentrations of peptides at the wound site are essential, together with evaluation of possible reductions in the peptide efficacy following modifications in the hydrogel network and optimization to promote the overall antimicrobial and repair properties of the hydrogels [90].

A combination of AMP-loaded nanoparticles and hydrogels have a great potential in preventing peptide degradation as well as in the increased time present in wound sites to promote overall antimicrobial and wound healing properties. Natural polymers that are components of the wound healing environment (e.g., collagen and hyaluronic acid) possess great potential in providing the on-demand spatiotemporally controlled release of AMP-loaded nanoparticles for wound healing [90].

Nanofiber functionalization with AMPs can provide both tissue repair and antimicrobial effects. The effectiveness of nanofibers in tissue repair is associated with their properties, such as porosity, air permeability, and surface wettability. The applications of electrospun nanofibers include suture coating, dermal replacements, and skin regeneration matrices. After the incorporation of AMPs, they can retain their antimicrobial activity and provide local controlled release with strong antibacterial effects [89].

The antimicrobial peptide Cys-KR12, the synthetic derivative of LL-37, immobilized on silk fibroin nanofiber membranes is a model of wound dressing by electrospinning and it has shown multiple functions in wound healing processes, such as antimicrobial activity, facilitation of cell proliferation, keratinocyte differentiation, as well as inhibition of pro-inflammatory cytokine expression [91].

Loading peptides into solid lipid nanoparticles and lipid vesicles can have several advantages, such as decreased toxicity, protection against physical or chemical degradation, improved bioavailability, and a desirable release profile. The therapeutic use of these nanoparticles endowed with proteinase inhibitors decreased elastase activity in chronic wounds and accelerated healing by regulating the inflammatory response to prevent tissue injury and to increase their bactericidal potential [89].

As described above, different formulations including AMPs loaded in nanoparticles, hydrogels, creams, gels, ointments, and wafers have been developed for effective AMP delivery to the site of injury. Design of smart, stimuli-responsive, and local wound microenvironment-responsive formulations (e.g., protease responsive, ROS scavenging, synergistic combinations of peptides, pH-responsive, photo-responsive systems) can release the AMPs in a spatiotemporally controlled way to promote tissue repair and antimicrobial effects. Furthermore, the development of biocompatible and biodegradable formulations to promote immunomodulation, reduce toxicity, and enhance re-epithelialization and granulation tissue formation is necessary to obtain the best outcome for wound healing [90]. The relatively high production cost of AMPs is another reason that limits broad clinical applications of AMPs. Solid-phase peptide synthesis is the most commonly used method for peptide synthesis. The other AMP production methods include production systems (bacteria, yeast, insects, and mammalian cells) for recombinant peptides, which require a longer time, high cost, and make introducing structural modifications difficult. Therefore, improving the existing methods or seeking new peptide synthesis methods may be a potential way to reduce production costs. In addition, the use of ultrashort or truncated peptides is a promising solution, as the shorter amino acid sequences involved lower the synthesis cost [43].

Another problem is that the antimicrobial activity of AMPs is highly sensitive to physiological conditions in the organism, resulting in a difference between the in vitro activity and actual in vivo efficacy. When exposed to physiological conditions, many factors, such as interaction with host cells, binding with serum albumin, and physiological salt concentration, may greatly reduce the direct antibacterial effect of AMPs. Thus, it was noted that in vivo the antibacterial effect requires higher than physiological concentrations of AMPs, whereas immunomodulatory, antibiofilm, and wound healing properties are preserved under physiological concentrations. This paradox could be explained by degradation of AMPs due to their sensitivity to the wound environment or by proteolysis, resulting in a decrease in antibacterial activity but not in the immunomodulatory effect. Peptides that do not show any in vitro antibacterial effects can still clearly prevent infections and indirectly exert in vivo antibacterial activity through these immunomodulatory effects. Together, the difference between in vitro and in vivo efficacy hinders the clinical use of AMPs, and hence, the AMP formulation strategies and the underlying antibacterial mechanisms need further investigation. Additionally, despite a large number of publications reporting the wound healing activities of AMPs (such as promoting cell migration and proliferation, inducing angiogenesis, stimulating collagen synthesis, and wound contraction), most of the studies are carried out in mice models, in which wound healing is mainly caused by contraction rather than re-epithelialization and granulation tissue formation. Thus, the investigation of specific wound healing activities of AMPs is limited to the models used. Therefore, because some AMPs have shown high activities in pig and rabbit wound healing models, these preclinical models should be considered for further in vivo experiments [43].

To address these challenges, an AMP crude neutrophil extract was prepared and evaluated as an antimicrobial, stable compound with the potential to activate immune cells. The obtained results are only preliminary and require a thorough clinical trial. Porcine blood, containing numerous components that have been extensively studied for their structure, mechanism of action, and activity against pathogens, as well as blood from other animal species, are potential sources of promising therapeutic agents [28,60]. In addition to their antimicrobial properties, these mixtures have numerous immunomodulatory effects [28,41,92].

Apart from these approaches, researchers are attempting to find new cationic peptides or modify natural products in order to obtain more effective and safer antibacterial peptides as potential therapeutics. Unfortunately, the development of satisfactory products is time-consuming and very costly. Nevertheless, AMPs hold great potential and have a promising future [17,25].

## 7. Conclusions

Among the many therapeutic options, some preparations, such as PRP, have been introduced into clinical practice, while others are waiting for their chance. Due to its easy access and versatility, porcine neutrophil crude extract appears to be a promising blood-derived product in regenerative medicine. Given the limitations in AMPs’ use, including AMPs of both of natural and synthetic origin, it should be emphasized that AMP extract lyophilizates can be manufactured at low cost and stored for a long time. This warrants further research into the potential applications of this preparation in regenerative medicine. Other neutrophil-derived products, specifically DGPs and MVs, may also hold promise as therapeutic agents for a number of diseases. This is due to their potential to activate immune cells, especially macrophages and neutrophils, which is essential to produce an adequate protective effect and to enhance the repair process by acting on immune cells. After preliminary studies, these products require thorough clinical trials before being introduced into practice. However, the experiments performed to date have delivered promising results. This means that a new therapeutic strategy, relying on both the antimicrobial and immunomodulatory properties of blood-derived products, will soon be available for inflammatory diseases, delayed bone repair, and biomaterial-associated infections.

## Figures and Tables

**Table 1:** Advantages and disadvantages of AMPs for therapeutic applications in regenerative medicine.

Advantages	Disadvantages
Broad-spectrum of antimicrobial activities Low bactericidal or antibacterial concentration (at microscale or nanoscale range) Pro-angiogenic and wound healing properties Immunomodulatory and anti-inflammatory properties Good safety and tolerability profile because amino acids are metabolites Low potential to bacterial resistance development Potential for inhibition of the formation of biofilms on chronic wounds and medical devices The synergistic action with antibiotics to improve the antibacterial effect Potential for application in flexible and changeable formulations, such as encapsulation into different delivery systems, self-assembly, and conjugation with polymers	Short half-life and susceptibility to proteolysis, leading to loss of biological activities. Possible hemolytic and cytotoxic effect to host cells at high concentration Sensitivity to physiological conditions, and activities may be inhibited by factors such pH, low concentration of salt, etc. Possible development of cross-resistance to certain AMPs by some antibiotic-resistant bacterial strains Small number of AMPs approved by regulatory agencies for use due to poor performances of AMPs in clinical trails Limited data about side effects, limited toxicology datasets Not standardized experimental conditions and evaluation criteria of AMPs activities Limited long-term stability for storage in ambient conditions High costs of AMPs production and purification, limited number of manufacturers that can produce peptides on a commercial scale

**Table 2:** Selected AMPs for regenerative applications that are in clinical trials or approved, to 2020.

Peptide	Indication	Indication	Status
CLS001 (omiganan, MBI226) Topical	Omiganan pentahydrochloride, synthetic cationic indolicidin derivative	Local catheter site infections	Phase III complete (discontinued)
		Topical skin antisepsis	Phase III complete
		Papulopustular rosacea	Phase III current
		Acne vulgaris	Phase II complete
		Atopic dermatitis	Phase II complete
		Vulvar intraepithelial neoplasia	Phase II complete
		Condylomata acuminata (external genital warts)	Phase II complete
		Facial seborrhoeic dermatitis	Phase II current
Iseganan (IB-367) Topical	Analogue of protegrin 1	Ventilator-associated pneumonia	Phase II/III; rejected, no efficacy
Iseganan (IB-367) Oral		Oral mucositis in patients with head and neck cancer	Phase III complete; no efficacy
PXL01 Topical	Synthetic macrocyclic 25-amino acid peptide derived from human lactoferricin	Prevention of postsurgical adhesions and scar prevention	Phase IIb complete; phase III trials planned
LL-37 Topical	Human cathelicidin subunit	Venous leg ulcers	Phase IIb current
Brilacidin (PMX-30063) Topical	Synthetic defensin mimetic	Ulcerative proctitis/ulcerative proctosigmoiditis	Phase II complete; phase III planned
		Oral mucositis in patients with head and neck cancer	Phase II complete; FDA fast track designation
Brilacidin (PMX-30063) Intravevenous	Synthetic defensin mimetic	Acute bacterial skin and skin structure infections	Phase II complete; phase III planned; FDA fast track designation
Murepavadin (POL7080) Intravenous	Synthetic cyclic b-hairpin Peptidomimetic based on the cationic antimicrobial peptide protegrin I	Ventilator- associated bacterial pneumonia caused by Pseudomonas aeruginosa	Phase III; suspended, adverse events
Neuprex, opebacan, BPI rBPI21 Intravenous	BPI-derived peptide	Burns	Phase II complete
		Myeloablative allogeneic hematopoietic stem cell transplantation	Phase I/II; terminated, lack of enrolment
hLF1-11 Intravenous	First cationic domain of human lactoferrin (11 residues)	Infections during hematopoietic stem cell transplantations	Phase I/II complete; withdrawn
		Candidaemia	Phase I/II; withdrawn
		Bacteremia due to Staphylococcus epidermidis	Phase I/II; withdrawn
